# New York Letter

**Published:** 1896-04

**Authors:** 


### NEW YORK LETTER.

NEW YORK, March 2, 1896.

The world has not become hysterical over the Roentgen rays and the new photography, but a lively interest in it is taken on all sides, and the newspapers perhaps have published much that is silly, unscientific and ignorant. The scientific world has laid aside much of its work to study its possibilities, and the medical world is alert to make practical use of it, should it be proven that its use is practical.

All that has come to us from the medical journals, and every New York journal has its Roentgen article, shows that as yet it is only of scientific interest, and that only the hand or foot can be photographed, forming a very shadowy outline with the bones in a little deeper shade, and these are the only tissues which have any distinctive features from the rest. So that a fracture or possibly bone disease or the presence of a foreign body is all that can be determined by it, which, interesting as it may be scientifically, can be of but little practical benefit, consequently the New York medical world is willing to bide its time.

Perhaps the orthopedists will be able to make use of it in the study of joint diseases, and if the idea suggested by one enthusiast prove true that the X rays possess germicidal qualities and may perhaps be used to kill the germs *in situ*, what a delightful dream for the orthopedist, the mere photographing of his hip joint cases in order to study their development may at the same time be the therapeutic agent which brings them back to health.

The new St. Luke's Hospital at Morningside Heights was opened early in February to receive the few patients remaining in the old building and doubtless will soon be running with its full quota of patients. The old building is being rapidly removed and some of the new dwelling houses to be built on its site have been begun.

Dr. John S. Billings has accepted the position of superintendent-in-chief of the Astor, Lenox and Tilden libraries, which have been consolidated, and the trustees are indeed fortunate that they may avail themselves of his able services.

The surgical section of the Academy of Medicine re-elected its chairman of last year, Dr. B. Farquhar Curtis, and for secretary elected Dr. John B. Walker. The genito-urinary section elected Dr. Wm. K. Otis for chairman, and Dr. Ramon Guiteras for secretary.

At the February meeting of the Surgical Section a paper was read by Dr. D. H. Goodwillie, entitled "Amputation of the Tongue for Malignant Disease by Electro-Surgical Means." The author read a short paper on the differential diagnosis, and exhibited an electric ecraseur and electric cautery knives for performance of the operation. The general sense of the meeting was that the proceeding was unsurgical; that the results by this method were notoriously bad; that it did not promise proper eradication of the disease, in that infected glands were left behind; further, that a painful wound was left in the mouth, which it was difficult to keep clean, and was long in healing, while by the Kocher method of removal of the tongue, a clean wound was made; the mucous membrane sutured, it required but little care and healed rapidly. Dr. Goodwillie admitted that the patients operated on by his method did not live long, but he thought their lives were prolonged a year or more by removal of the malignant growth by his method.

Dr. W. B. Coley, at the same meeting, presented a boy who had been operated on at the Post-graduate Hospital for appendicitis of traumatic origin. Three days before coming to the hospital he had been struck by a push cart, and he presented himself to be fitted with a truss, and the true diagnosis was made, and the boy operated on at midnight; the appendix was gangrenous, and there was an abscess. Dr. Coley also presented a baby, three months old, with a patent urachus, stating that this was the only case of the kind that had been seen at the Hospital for five years. There was some enlargement of the umblicus, and constant dribbling of urine from the opening. Dr. Coley thought it probable that it would heal without operation, but that if it did not in two or three years, then an operation would be undertaken for its closure.

